# Low gamma-glutamyl transpeptidase levels at presentation are associated with severity of liver illness and poor outcome in biliary atresia

**DOI:** 10.3389/fped.2022.956732

**Published:** 2022-09-23

**Authors:** Song Sun, Shan Zheng, Chun Shen, Rui Dong, Kuiran Dong, Jingying Jiang, Yifan Yang, Gong Chen

**Affiliations:** Surgical Department, Children’s Hospital of Fudan University, Shanghai, China

**Keywords:** biliary atresia, gamma-glutamyl transpeptidase (GGT), liver function, jaundice clearance, prognosis

## Abstract

**Objective:**

To investigate the clinical features and prognosis of biliary atresia (BA) with normal or minimally elevated gamma-glutamyl transpeptidase (GGT).

**Methods:**

The clinical data of patients with BA in our hospital between 2012 and 2017 were retrospectively studied. The patients were divided into a low-GGT group (GGT ≤ 300 IU/L) and a high-GGT group (GGT > 300 IU/L) according to the preoperative GGT level. The perioperative clinical parameters, the postoperative jaundice clearance within 6 months, and the 2-year native liver survival were compared among the groups.

**Results:**

A total of 1,998 children were included in this study, namely, 496 in the low-GGT group and 1,502 in the high-GGT group. The ages and weights at the surgery in the low-GGT group were significantly lower than those in the high-GGT group (64.71 ± 21.35 vs. 68.64 ± 22.42 days, *P* = 0.001; 4.67 ± 1.03 vs. 4.89 ± 0.98 kg, *P* < 0.001). The levels of serum ALP, ALT, and AST in the low-GGT group were significantly higher than those in the high-GGT group before and 2 weeks after the surgery (ALP: 647.52 ± 244.10 vs. 594.14 ± 228.33 U/L, *P* < 0.001; ALT: 119.62 ± 97.14 vs. 96.01 ± 66.28 U/L, *P* < 0.001; AST: 218.00 ± 173.82 vs. 160.71 ± 96.32 U/L; *P* < 0.001). The INR of the low-GGT group was higher than that of the high-GGT group (1.05 ± 0.34 vs. 0.98 ± 0.20, *P* < 0.001), while FIB was lower than the high-GGT group (2.54 ± 0.67 vs. 2.73 ± 1.44 g/L; *P* = 0.006). The decreasing amplitude of TB and DB within 2 weeks after surgery in the low-GGT group was smaller than those in the high-GGT group (TB: 51.62 ± 71.22 vs. 61.67 ± 53.99 μmol/L, *P* = 0.003; DB: 33.22 ± 35.57 vs. 40.20 ± 35.93 μmol/L, *P* < 0.001). The jaundice clearance rate in the low-GGT group was significantly lower than that in the high-GGT group at 1, 3, and 6 months after surgery (17.70 vs. 26.05%; 35.17 vs. 48.58%; 38.62 vs. 54.64%, *P* < 0.001). In addition, the 2-year native liver survival rate in the low-GGT group was significantly lower than that of the high-GGT group (52.5 vs. 66.3%, *P* < 0.001 HR 1.80, 95% CI 1.38–2.33).

**Conclusion:**

Compared to patients with high GGT, patients with normal or minimally elevated pre-operative GGT in BA were found to have poorer pre-operative liver function parameters, and post-operatively had lower jaundice clearance rates and worse 2-year native liver survival. This suggests a lower GGT at presentation in biliary atresia could be a sign of more severe liver injury.

## Introduction

The serum level of gamma-glutamyl transpeptidase (GGT) in most patients with biliary atresia (BA) is significantly increased, which plays an important role in the differential diagnosis of neonatal cholestatic disorders (1–7). However, some neonates initially presenting with normal or minimally elevated GGT levels are eventually, and sometimes unexpectedly, confirmed to have BA (8, 9). Unfortunately, this may lead to delayed diagnosis and treatment. In addition, the postoperative prognosis of patients with lower GGT is understudied, with only a few studies suggesting that lower preoperative GGT may be associated with poor prognosis (10, 11). Due to the low incidence of BA with normal or minimally elevated GGT, the previous studies on the correlation between low GGT and prognosis were mostly based on small cohorts (11, 12). In this study, the clinical data of nearly 2,000 patients with BA diagnosed and treated in a single center within 6 years were analyzed and the clinical characteristics and prognosis of patients with normal or minimally elevated GGT were studied.

## Materials and methods

Chart reviews were conducted on infants diagnosed with BA between 1 January, 2012, and 31 December, 2017, at the Children’s Hospital of Fudan University. The diagnosis of BA was confirmed through surgical biliary exploration and/or cholangiography. Demographics (age, sex, and body weight), and also preoperative and postoperative laboratory data (transaminases, liver function, and coagulation markers) were collected. This study was approved by the Ethics Committee of the Children’s Hospital of Fudan University. We reviewed several studies on the diagnostic efficacy of GGT in differentiating BA from other cholestatic diseases and found that the threshold values in most studies were between 250 and 300 IU/L (1, 4, 6, 13–15). Therefore, 300 IU/L was used as the critical cut-off value differentiating the low-GGT group (≤300 IU/L) from the high-GGT group (>300 IU/L) based on the preoperative GGT level. The patients underwent Kasai procedures (KPs) between July 2014 and December 2017 and were followed up through clinic or telephone interviews. Clearance of jaundice (COJ) within 6 months and native liver survival rates within 2 years were recorded for prognostic evaluation. Clearance of jaundice was defined as total bilirubin (TB) < 20 mmol/L (11, 16). The correlation between preoperative GGT level and prognosis was analyzed.

The SPSS 21 software package was used for statistical analysis. Measurement data were first tested for normality. A *t*-test was used for variables with normal distribution, and a non-parametric test was used for variables without normal distribution. The chi-square test was used for categorical variables such as sex ratios and survival rates, COX regression was used in survival analysis. *P*-value less than 0.05 was considered a significant difference.

## Results

### Demographics

**FIGURE 1:**
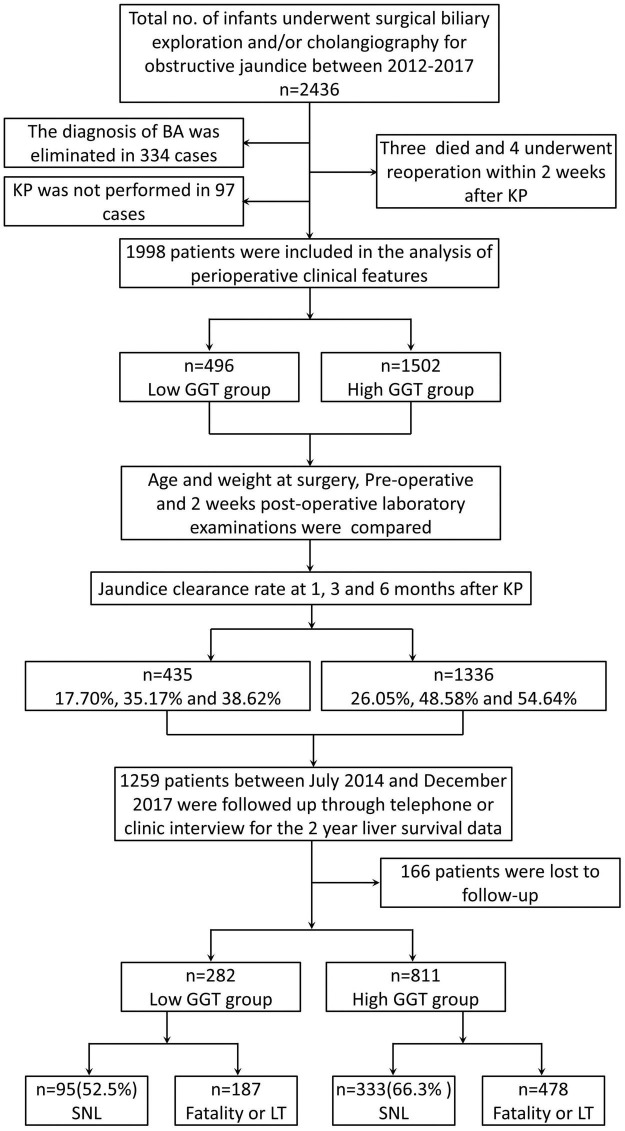
Flowchart showing the study population and their outcomes. BA, biliary atresia; KP, Kasai procedure; GGT, gamma-glutamyl transpeptidase; SNL, survival with native liver; LT, liver transplantation.

A total of 2,436 children with obstructive jaundice underwent surgical biliary exploration and/or cholangiography in our hospital between 1 January, 2012, to 31 December, 2017. In total, 2,095 were confirmed to have BA. A total of 1,998 patients underwent the Kasai procedure (KP) and were included in this study (Figure 1). There were 496 cases sub-grouped in the low-GGT group, including 245 men, and 1,502 cases in the high-GGT group, including 769 men. There was no statistical difference in the gender composition ratio between the two groups (*P* > 0.05). The mean age and weight at surgery in the low-GGT group were both significantly lower than that in the high-GGT group (64.71 ± 21.35 vs. 68.64 ± 22.42 days, *P* = 0.001 and 4.67 ± 1.03 vs. 4.89 ± 0.98 kg, *P* < 0.001, respectively) (Table 1).

**TABLE 1:** Comparison of preoperative clinical indicators between low and high GGT patients.

	Low GGT BA	High GGT BA	*P*-value
N	496	1,502	
Gender (Male)	245	769	0.486
Age at surgery (days)	64.71 ± 21.35	68.64 ± 22.42	0.001*
Weight at surgery (Kg)	4.67 ± 1.03 Kg	4.89 ± 0.98	0.000*
**Preoperative liver function**			
TB (μmol/L)	165.72 ± 55.58	166.42 ± 55.43	0.807
DB (μmol/L)	111.66 ± 39.16	115.16 ± 35.34	0.066
ALP (U/L)	647.52 ± 244.10	594.14 ± 228.33	0.000*
ALT (U/L)	119.62 ± 97.14	96.01 ± 66.28	0.000*
AST (U/L)	218.00 ± 173.82	160.71 ± 96.32	0.000*
ALB (g/L)	38.64 ± 3.60	38.84 ± 3.59	0.291
TBA (μmol/L)	133.37 ± 62.40	138.30 ± 54.21	0.015*
**Preoperative coagulation function**			
APTT (s)	44.63 ± 7.05	41.80 ± 6.99	0.000*
PT (s)	13.47 ± 2.59	13.07 ± 3.72	0.029*
TT (s)	18.73 ± 4.80	18.62 ± 4.55	0.663
FIB (g/L)	2.54 ± 0.67	2.73 ± 1.44	0.006*
INR	1.05 ± 0.34	0.98 ± 0.20	0.000*
**Classification**			
BA with malformations (*n* = 401)	114 (22.98%)	287 (19.11%)	0.062
BASM (*n* = 58)	15 (3.02%)	43 (2.86%)	0.853
CMV-IgM + ev associated BA	132/395 (33.42%)	496/1,191 (41.51%)	0.004*

GGT, gamma-glutamyl transpeptidase; BA, biliary atresia; TB, total bilirubin; DB, direct bilirubin; ALP, alkaline phosphatase; ALT, alanine aminotransferase; AST, aspartate aminotransferase; TBA, total bile acid; ALB, albumin; APTT, activated partial thromboplastin time; PT, prothrombin time; TT, thrombin time; FIB, fibrinogen; INR, international normalized ratio; BASM, biliary atresia splenic malformation. *p < 0.05.

### Preoperative liver function and coagulation markers

There were no significant differences in preoperative TB, direct bilirubin (DB), and albumin ALB among the 2 groups (165.72 ± 55.58 vs.166.42 ± 55.43 μmol/L, *P* = 0.807; 111.66 ± 39.16 vs. 115.16 ± 35.34 μ/L, *P* = 0.066; 38.64 ± 3.60 vs. 38.84 ± 3.59 g/L, *P* = 0.291), while alkaline phosphatase (ALP), alanine aminotransferase (ALT), and aspartate aminotransferase (AST) in the low-GGT group were significantly higher than those in the high-GGT group (647.52 ± 244.10 vs. 594.14 ± 228.33 U/L, *P* < 0.001; 119.62 ± 97.14 vs. 96.01 ± 66.28 U/L, *P* < 0.001; 218.00 ± 173.82 vs. 160.71 ± 96.32 U/L, *P* < 0.001), total bile acid (TBA) in the low-GGT group was lower than that in the high-GGT group (133.37 ± 62.40 vs. 138.30 ± 54.21 μ/L, *P* = 0.015). In terms of coagulation markers, APTT, PT, and TT were all significantly prolonged in both groups. However, prolongation of APTT and PT was statistically higher in the low-GGT group vs. the high-GGT group (44.63 ± 7.05 vs. 41.80 ± 6.99 s, *P* < 0.001; 13.47 ± 2.59 vs. 13.07 ± 3.72 s, *P* = 0.029). The FIB in the low-GGT group was lower than that in the high-GGT group (2.54 ± 0.67 vs. 2.73 ± 1.44 g/L, *P* = 0.006), while INR was statistically significantly higher in the low-GGT group vs. the high-GGT group (1.05 ± 0.34 vs. 0.98 ± 0.20, *P* < 0.001) (Table 1).

### CMV–IgM positivity and BA with splenic malformation

According to the preoperative examination and surgical exploration, a total of 401 patients were diagnosed as BA with malformations, of which 58 were BA with splenic malformation (BASM). Of those infants diagnosed with BA with malformations, 114 were classified into the low-GGT group, the other 287 cases were classified into the high-GGT group. They accounted for 22.98 and 19.11% of each group, respectively (χ^2^ = 3.492, *P* = 0.062). Of the 58 infants diagnosed with BASM, 15 were classified into the low-GGT group and 43 into the high-GGT group, accounting for 3.02 and 2.86% of each group (χ^2^ = 0.034, *P* = 0.853).

The CMV–IgM test was performed in 1,586 children, the positive rate was 33.42% in the low-GGT group, and 41.51% in the high-GGT group. The positive rate of CMV–IgM in the low-GGT group was significantly lower than that in the high-GGT group (χ^2^ = 8.396, *P* = 0.004).

### Jaundice clearance rates

The average TB and DB decreased significantly in both groups 2 weeks after surgery (TB decrease: 51.62 ± 71.22 vs. 61.67 ± 53.99 μ/L, *P* = 0.003; DB decrease: 33.22 ± 35.57 vs. 40.20 ± 35.93 μmol/L, *P* = 0.001). The TB and DB at 2 weeks after surgery in the low-GGT group, with a milder decrease, were higher than those in the high-GGT group (116.14 ± 68.25 vs. 103.99 ± 47.03 μmol/L, *P* < 0.001; 80.06 ± 32.46 vs. 74.62 ± 32.26 μmol/L, *P* = 0.003). The ALP, ALT, and AST all decreased to various degrees 2 weeks after the operation, but were still statistically significantly higher in the low-GGT group than those in the high-GGT group (459.96 ± 209.53 vs. 415.98 ± 167.61 IU/L, *P* < 0.001; 108.95 ± 79.50 vs. 85.40 ± 55.82 U/L, *P* < 0.001; 160.26 ± 96.14 vs. 117.85 ± 63.06 U/L, *P* < 0.001), which were consistent with the preoperative difference. However, there was no significant difference in the decrease ratio of ALP, ALT, and AST between the two groups (186.35 ± 221.38 vs. 179.74 ± 192.28 IU/L, *P* = 0.559; 11.78 ± 80.64 vs. 13.46 ± 78.61 U/L, *P* = 0.804; 57.04 ± 142.83 vs. 44.40 ± 96.89 U/L, *P* = 0.102) (Table 2).

**TABLE 2:** The main indicators of liver function 2 weeks after operation and their decrease compared to preoperative levels in the low and high-GGT groups.

	Low-GGT group	High-GGT group	*P*-value	Decrease in low-GGT group	Decrease in High-GGT group	*P*-value
TB (μmol/L)	116.14 ± 68.25	103.99 ± 47.03	0.000*	51.62 ± 71.22	61.67 ± 53.99	0.003*
DB (μmol/L)	80.06 ± 32.46	74.62 ± 32.26	0.003*	33.22 ± 35.57	40.20 ± 35.93	0.001*
ALP (1U/L)	459.96 ± 209.53	415.98 ± 167.61	0.000*	186.35 ± 221.38	179.74 ± 192.28	0.559
ALT (U/L)	108.95 ± 79.50	85.40 ± 55.82	0.000*	11.78 ± 80.64	13.46 ± 78.61	0.804
AST (U/L)	160.26 ± 96.14	117.85 ± 63.06	0.000*	57.04 ± 142.83	44.40 ± 96.89	0.102

GGT, gamma-glutamyl transpeptidase; TB, total bilirubin; DB, direct bilirubin; ALP, alkaline phosphatase; ALT, alanine aminotransferase; AST, aspartate aminotransferase. *p < 0.05.

The TB and DB were tested at 1, 3, and 6 months after KP, respectively. Complete data for TB and DB at all three-time intervals were available for 1,771 children, namely, 435 in low-GGT group and 1,336 in high-GGT group. The rates of COJ were 17.70, 35.17, and 38.62% in the low-GGT group, and 26.05, 48.58, and 54.64% in the high-GGT group at 1, 3, and 6 months post-operation, respectively, statistically significantly lower in the low-GGT group than the high-GGT group (χ^2^ = 12.535, *P* < 0.001; χ^2^ = 23.800, *P* < 0.001; χ^2^ = 33.694, *P* < 0.001).

### Two-year native liver survival

**FIGURE 2:**
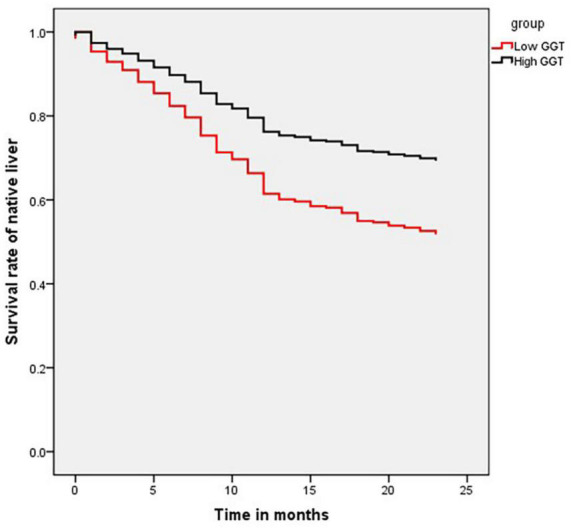
Kaplan–Meier curve showing native liver survival of low- and high-GGT groups.

In total, 1,259 patients who underwent KP between July 2014 and December 2017, were followed up through telephone or clinic interviews, of which 1,093 (282 in low-GGT group and 811 in high-GGT group) were successfully followed up for more than 2 years. The 2-year native liver survival rate was 52.5% in the low-GGT group vs. 66.3% in the high-GGT group (*P* < 0.001 HR 1.80, 95% CI 1.38–2.33) (Figure 2). In the multivariate Cox-regression model, in addition to GGT level, age at surgery was also determined to be an independent risk factor for native liver survival (*P* < 0.001 HR 1.02, 95% CI 1.01–1.03), weight at surgery, pre-operative TB, DB, ALP, ALT, AST, TBA, APTT, PT, FIB, INR, and CMV–IgM + were not correlated with the 2-year native liver survival (Table 3).

**TABLE 3:** The multivariate Cox-regression analysis of independent risk factors for native liver survival.

Variable	*P*-value	HR (95% CI)
GGT grouping	0.000	1.796 (1.384–2.329)
Age at surgery	0.000	1.019 (1.013–1.026)
Weight at surgery	0.120	1.092 (0.977–1.220)
TB	0.693	1.001 (0.995–1.007)
DB	0.721	1.002 (0.993–1.010)
ALP	0.992	1.000 (0.999–1.001)
ALT	0.235	0.999 (0.996–1.001)
AST	0.389	1.001 (0.999–1.002)
TBA	0.460	1.001 (0.999–1.002)
APTT	0.325	1.011 (0.989–1.034)
PT	0.329	0.970 (0.914–1.031)
FIB	0.628	1.047 (0.868–1.264)
INR	0.723	1.104 (0.638–1.911)
CMV-IgM+	0.488	0.919 (0.723–1.168)

GGT, gamma-glutamyl transpeptidase; BA, biliary atresia; TB, total bilirubin; DB, direct bilirubin; ALP, alkaline phosphatase; ALT, alanine aminotransferase; AST, aspartate aminotransferase; TBA, total bile acid; APTT, activated partial thromboplastin time; PT, prothrombin time; TT, thrombin time; FIB, fibrinogen; INR, international normalized ratio; CMV, cytomegalovirus.

## Discussion

The significant GGT elevation in BA has been widely cognized and used in screening and diagnosis of BA (6, 7, 14, 17–19). Patients with normal or minimally elevated GGT are vulnerable to being delayed in the diagnosis. These patients should have a longer waiting time and an older age at surgery theoretically, because of more examinations and longer clinical observation before surgery. However, the results of this study showed that patients in the low-GGT group were younger and had lower weight at the time of surgery, which was contrary to the expectations. Previous studies have reported that the GGT of BA increases gradually with the age of the patient (6, 14, 20). The lower GGT might be correlated with younger age, and BA with lower GGT than expected may have earlier onset. Children of younger age, presently with lower-than-expected GGT in BA, theoretically may actually be presenting earlier because of more aggressive disease with more significant liver illness (21). The comparison of liver function and coagulation markers in this study also suggested that patients with BA with lower GGT present with more advanced liver disease. Earlier onset and more severe illness, which lead to earlier medical treatment in these children might account for the difference in age and weight.

At present, there is a paucity of literature documenting the correlation between low GGT and the severity of liver injury in BA. However, reports exist suggesting low or normal GGT is correlated with the severity of liver illness in other neonatal liver diseases. Oswari et al. (22) conducted a retrospective study of 181 neonates with cholestasis associated with neonatal septicemia and found that low GGT was associated with more severe disease. Wang et al. (21) analyzed the GGT levels in 103 cases of idiopathic neonatal hepatitis and found that bilirubin and transaminases were higher, and histologically, liver injuries were more severe in patients with low and normal GGT. The results of our study showed that preoperative transaminitis and coagulopathy were worse in patients with normal to minimally elevated GGT, suggesting low-preoperative GGT levels in BA may be correlated with more severe liver injury. The molecular mechanism of this correlation is still unknown. The GGT catalyzes the transfer of the glutamyl moiety from glutathione to restore intracellular glutathione levels (23), and the deficiency of GGT may aggravate oxidative stress damage in the liver. Luo et al. (24) reported in a recent study suggesting the expression of genes regulating glutathione metabolism were unregulated in children with BA who survived more than 2 years with the native liver. Injection of N-acetylcysteine could reduce liver injury and fibrosis in a mouse model of BA (24), which further hypothesized the important role of glutathione in BA against oxidative stress. Therefore, lower GGT may lead to decreased ability to respond to oxidative stress injury, thus aggravating liver injury (25). In addition, glutathione had been shown to drive bile acid transport (26). However, the results of this study showed that the TBA in the low-GGT group was lower than that in the high-GGT group, which is inconsistent with this conclusion. The intrinsic correlation between GGT and bile acid in BA requires further study.

As a matter of interest, CMV-IgM positivity in the low-GGT group was significantly lower than that in the high-GGT group. The correlation between CMV-IgM positivity and GGT level in BA has not been reported previously. Since the role of CMV infection in the pathogenesis of BA is still unclear, the intrinsic association between CMV infection and GGT level is also unknown. Based on the results of this study, jaundice appeared early in patients with lower GGT, which may result in more or earlier interruption of breastfeeding, and reducing the probability of postpartum CMV breastmilk transmission. This might be the reason for the low positivity rate of CMV–IgM in the low-GGT group. In addition, in the series reported from western countries, about 10% of the BA patients were CMV–IgM + ve (27). In contrast, BA with CMV–IgM + ve appears to be more frequent among affected Asian infants (28, 29). Xu et al. has reported that up to 50% of BA cases in China could be CMV related based on a study of liver biopsies (29). The high incidence of CMV–IgM positive associated biliary atresia may be an epidemiological feature unique to eastern countries (30).

The prognosis of BA with lower GGT was rarely reported previously. The effect of poorer pre-operative liver function on prognosis was also still unknown. In our previous study (10), we compared the preoperative GGT levels of BA patients who survived for more than 5 years with the native liver and normal liver function, with the patients who suffered liver failure within 1 year after KP. Results showed that the preoperative GGT levels of children with good prognosis were significantly higher than those with poor prognosis. Shankar et al. (11) reported 113 children who underwent Kasai, grouped into normal GGT vs. the high-GGT group using 200 IU/L as the critical value, and statistically found that 12.3% of children with BA had GGT lower than 200 IU/L. Children with normal GGT levels received liver transplantation at a younger age and had a significantly lower native liver survival rate. Zhang et al. (31) analyzed the correlation between preoperative GGT and postoperative COJ in 175 patients and found that low GGT was an independent risk factor for failure of postoperative COJ. As a whole, these results suggest that lower preoperative GGT levels may be associated with poor prognosis after KP.

The present study also showed that the recovery of liver function in patients with lower preoperative GGT was worse than that with higher preoperative GGT 2 weeks after surgery. The postoperative COJ rate at 1, 3, and 6 months, and the 2 years native liver survival rate were lower than that in the high-GGT group. The results of our study showed that the short- and long-term prognosis of BA in the low-GGT group was poorer, which was consistent with the results of previous studies. In addition, a multivariate survival analysis was conducted on preoperative indicators showing a statistically significant disadvantage to native liver survival in infants presenting with low or minimally elevated GGT compared to their high-GGT counterparts (older age at surgery being the only other identifiable independent risk factor). Considering the younger age in the low-GGT group was conducive to native liver survival, the interference of age as a confounding on the above conclusion could be eliminated, which further suggested the negative correlation between low-GGT and native liver survival.

Since our center did not establish a standardized follow-up system before June 2014, the rate of lost to follow-up was high before then. Therefore, these patients were excluded from the statistics of long-term prognosis, which may cause bias to the results. All the patients were generally divided into low- and high-GGT group in this study, while the potential confounding factors such as premature birth, birth weight, preoperative medication, and pathological classification were not fully investigated. In addition, only the COJ and native liver survival rates were adopted to assess the prognosis in this study. A detailed investigation of survival status (namely, liver function, liver stiffness, and esophagogastric variceal hemorrhage) of patients with native liver among the two groups can be more convincing for the conclusion. Nevertheless, due to the large sample size of this study, the results are still highly reliable. Given the severe illness and poor prognosis, the patients with lower preoperative GGT should be followed up closely and given higher priority on the waiting list for the liver transplantation. Further studies on liver pathology of patients with lower GGT might be beneficial to improve cognition of these patients.

In conclusion, a low-GGT level of BA may be associated with early onset, severe liver illness, and poor prognosis in the short and long term after KP. The molecular biological mechanism of the effect of low GGT on the pathophysiological process in BA remains to be further studied.

## Data availability statement

The raw data supporting the conclusions of this article will be made available by the authors, without undue reservation.

## Ethics statement

This study was approved by the Ethics Committee of the Children’s Hospital of Fudan University.

## Author contributions

SZ, GC, CS, RD, and KD: study conception and design. SS, JJ, and YY: data acquisition. SS and JJ: analysis and data interpretation. SS: drafting of the manuscript. SZ, GC, and RD: critical revision. All authors contributed to the article and approved the submitted version.
